# Artificial Intelligence Supporting the Training of Communication Skills in the Education of Health Care Professions: Scoping Review

**DOI:** 10.2196/43311

**Published:** 2023-06-19

**Authors:** Tjorven Stamer, Jost Steinhäuser, Kristina Flägel

**Affiliations:** 1 Institute of Family Medicine University Hospital Schleswig-Holstein Luebeck Campus Luebeck Germany

**Keywords:** communication, education, artificial intelligence, machine learning, health care, skill, use, academic, students, training, cost, cost-effective, health care professional

## Abstract

**Background:**

Communication is a crucial element of every health care profession, rendering communication skills training in all health care professions as being of great importance. Technological advances such as artificial intelligence (AI) and particularly machine learning (ML) may support this cause: it may provide students with an opportunity for easily accessible and readily available communication training.

**Objective:**

This scoping review aimed to summarize the status quo regarding the use of AI or ML in the acquisition of communication skills in academic health care professions.

**Methods:**

We conducted a comprehensive literature search across the PubMed, Scopus, Cochrane Library, Web of Science Core Collection, and CINAHL databases to identify articles that covered the use of AI or ML in communication skills training of undergraduate students pursuing health care profession education. Using an inductive approach, the included studies were organized into distinct categories. The specific characteristics of the studies, methods and techniques used by AI or ML applications, and main outcomes of the studies were evaluated. Furthermore, supporting and hindering factors in the use of AI and ML for communication skills training of health care professionals were outlined.

**Results:**

The titles and abstracts of 385 studies were identified, of which 29 (7.5%) underwent full-text review. Of the 29 studies, based on the inclusion and exclusion criteria, 12 (3.1%) were included. The studies were organized into 3 distinct categories: studies using AI and ML for text analysis and information extraction, studies using AI and ML and virtual reality, and studies using AI and ML and the simulation of virtual patients, each within the academic training of the communication skills of health care professionals. Within these thematic domains, AI was also used for the provision of feedback. The motivation of the involved agents played a major role in the implementation process. Reported barriers to the use of AI and ML in communication skills training revolved around the lack of authenticity and limited natural flow of language exhibited by the AI- and ML-based virtual patient systems. Furthermore, the use of educational AI- and ML-based systems in communication skills training for health care professionals is currently limited to only a few cases, topics, and clinical domains.

**Conclusions:**

The use of AI and ML in communication skills training for health care professionals is clearly a growing and promising field with a potential to render training more cost-effective and less time-consuming. Furthermore, it may serve learners as an individualized and readily available exercise method. However, in most cases, the outlined applications and technical solutions are limited in terms of access, possible scenarios, the natural flow of a conversation, and authenticity. These issues still stand in the way of any widespread implementation ambitions.

## Introduction

### Communication Skills in Health Care

In any health care profession, proficient communication skills are of great importance for the patient–health care provider relationship [1-5]. Effective communication has been directly associated with high patient satisfaction, a positive individual patient outcome [6,7], and a successful shared decision-making process [8]. In addition, the patient’s adherence to treatment [9] and adoptive preventive health behaviors [10] have been found to benefit substantially if they perceive their communication with the health care provider as pleasant.

Good communication skills can be acquired but need to be practiced continuously to be preserved [11,12]. To address both learning and maintaining these skills, communication skills training is provided in most health care education programs [13]. However, the study and practice of communication skills can be costly (eg, in terms of staff and materials) and time-consuming (eg, in terms of organization and scheduling) [14,15]. Furthermore, individuals who serve as actors for standardized patient–medical practitioner role-plays may experience feelings of anxiety, fatigue, or physical and mental discomfort [16,17]. Although these drawbacks have been reported to last for a short duration and have relatively few long-term negative consequences [18], technological advances such as artificial intelligence (AI), machine learning (ML), virtual reality, and virtual patients have become viable as supplements to traditional communication training for health professionals.

### Definitions of AI and ML

The exact definition of *AI* is still discussed [19,20]. In practice, the term is used as an umbrella term for all situations in which a system or machine makes a decision that requires some kind of *intelligence*. As vague as this definition may be, it also seems to be closely linked to the period it was used in [19,20]. Just a few years ago, even navigation systems and devices were considered AI. Today, if one says AI, one usually means autonomous vehicles, for instance. In a decade, it might mean something entirely different. Alternative definitions of AI often comprise the requirement that an *intelligent* system has to interact with its corresponding environment. Thus, the system has to be able to gather information about its periphery, act upon the gathered information, learn from it, and derive appropriate behavior from it [19,20].

*ML* is a subset of AI and is mostly used as a blanket term for all computer programs and machines that operate automatically and autonomously without being explicitly programmed to behave in a specific manner. How these programs and machines learn from data is predetermined, directing them toward a desired direction. As such, ML is a popular method of establishing a system that incorporates AI [19,20].

In this study, we used the aforementioned definitions of the 2 terms.

### Virtual Reality and Virtual Patients

The use of AI systems in health care is often accompanied by the use of virtual reality and virtual patients. Virtual reality was introduced to health care some time ago and has since been established as an effective means to counter symptoms of pain and anxiety [21,22]. It was also found to be associated with an improvement of gait and balance in the rehabilitation training of patients with Parkinson disease [23]. The use of virtual patients in simulations is a promising approach because these programs tend to possess a high degree of flexibility, are uniform, and can easily be standardized [24,25]. Furthermore, they are repeatable and practically tailorable to any given scenario and context [25,26]. In addition, simulated patient–medical practitioner conversations using virtual patients may add authenticity to the conversations in comparison with any analog or digital format that lacks a real or simulated face-to-face talk [26].

### AI and Communication Skills Training in Health Care

The aim of this scoping review was to evaluate how AI may support communication skills training in the academic training of health care professionals. Specifically, this review aimed to answer the following two questions: (1) How can AI support communication skills training for students pursuing health care profession education? and (2) What are the determinants (supporting and hindering factors) for the use of AI in communication skills training for students pursuing health care profession education?

## Methods

### Design and Search Strategy

This scoping review used the PRISMA-ScR (Preferred Reporting Items for Systematic Reviews and Meta-Analysis extension for Scoping Reviews) guidelines as well as the corresponding checklist (Multimedia Appendix 1) [27]. The protocol for this scoping review has not been registered or published.

A comprehensive literature search was performed in October 2022 using the PubMed, Scopus, Cochrane Library, Web of Science Core Collection, and CINAHL databases. The complete search strategy is presented in Multimedia Appendix 2. An update of this initial search was performed in February 2023.

### Ethical Considerations

As the scoping review focused on previously published literature and did not include any human participants, approval by an institutional review board was not required.

### Inclusion and Exclusion Criteria

Studies that covered either communication training per se or support for the improvement of communication skills training in health care profession education were included. In addition, to be included, these studies were required to comprise the use of either AI or ML. Furthermore, a detailed description of the corresponding application or use needed to be a part of the paper for it to be included. Owing to its educational setting, the study’s target population had to be undergraduate students pursuing health care profession education. Studies that targeted a population other than undergraduate health care students were excluded. Accordingly, papers that did not include communication skills training, academic health care professions, or the use of AI or ML and papers that lacked a description of the corresponding application were excluded. Reviews that met the inclusion criteria were screened for original research articles, which were then included instead of the reviews themselves.

### Screening

The screening process took place in two stages: (1) title and abstract screening and (2) full-text screening based on the aforementioned inclusion and exclusion criteria. In addition, reference lists of the retrieved full texts were hand searched for further eligible publications. The screening was performed by 2 independent researchers (TS and KF) who examined the studies in the first and the second stages. Any conflicts that arose were resolved through discussion and the intervention of a third researcher (JS) who acted as an independent supervisor. Upon reaching consensus, the respective publications were either included or excluded. The screening process was facilitated by using the Covidence web tool (Veritas Health Innovation) [28].

### Data Extraction Process

The aforementioned 2 independent screeners went on to extract the data from the included papers using a modified version of the Cochrane data collection form for intervention reviews: randomized controlled trials (RCTs) and non-RCTs (version 3). After careful consideration, the authors of this study agreed on using a modified version of the Cochrane data collection form owing to the heterogeneous nature of the included publications with respect to design and method. Any conflicts that arose were resolved through discussion until a consensus was reached. The extracted data included study characteristics (eg, the study’s aim, design, and unit of allocation), information about the participants or target group (if applicable, eg, population description, setting, and inclusion and exclusion criteria), the description of the intervention (eg, theoretical basis and the description of the intervention itself), and the study’s key conclusions.

Using an inductive approach, the included studies were organized into different categories. The categories were derived from the topics addressed in the included publications. The grouping of the articles was determined using a consensus approach.

We decided to provide information on the quality of evidence in the included papers because of the topicality of the research question [29,30]. Accordingly, and to provide recommendations for practice, we assigned the included studies to the levels of research evidence as established by Grove [31]. In this approach, level I represents the highest level of evidence, whereas level VII represents the lowest level of evidence.

## Results

### Paper Selection

**Figure 1:**
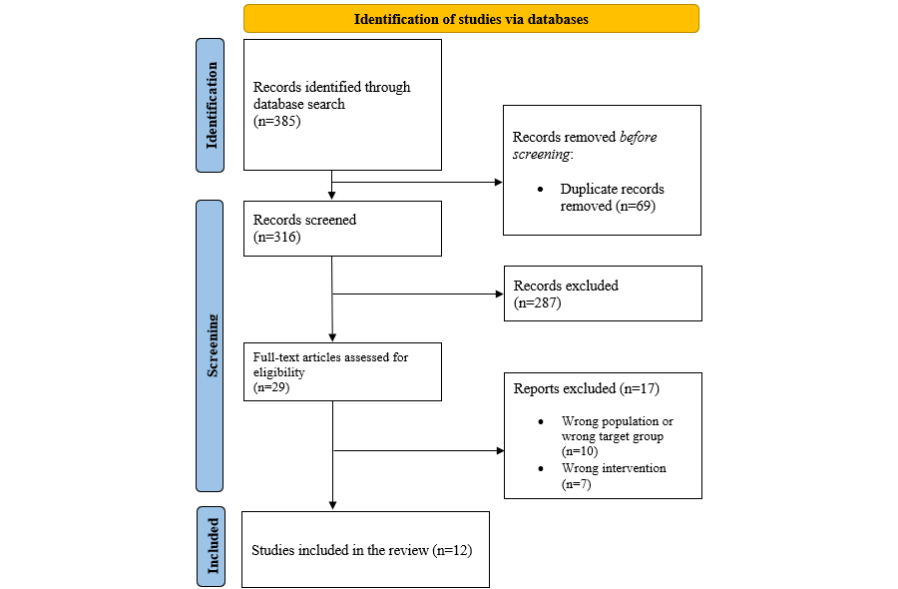
PRISMA (Preferred Reporting Items for Systematic Reviews and Meta-Analyses) flowchart for the identification of studies via databases.

The database searches identified a total of 385 publications. Upon removing 17.9% (69/385) duplicates, the titles and abstracts of 82.1% (316/385) of publications were screened. On the basis of the inclusion and exclusion criteria, the full texts of 7.5% (29/385) of publications were reviewed. In total, 3.1% (12/385) of studies were included in this scoping review (Figure 1).

### Thematic Categories

A frequency table provides an overview of the 3 final categories and sorting that was agreed upon (Table 1).

**Table 1:** Studies included in thematic categories.

Theme	Author, year of publication	Publications (n=12), n (%)
AI^a^ and ML^b^ for text analysis and information extraction	Jani et al [32], 2020	1 (8)
AI, ML, and virtual reality in communication skills training for students pursuing health care profession education	Liaw et al [33], 2023	1 (8)
AI, ML, and virtual patients in communication skills training for students pursuing health care profession education	Carnell et al [34], 2019; Furlan et al [35], 2021; Hamdy et al [36], 2017; Kobayashi et al [37,38], 2022; Maicher et al [39], 2019; Maicher et al [40], 2023; Shorey et al [41], 2019; Shorey et al [42], 2020; Shorey et al [43], 2023; Tavarnesi et al [44], 2018	10 (83)

^a^AI: artificial intelligence.

^b^ML: machine learning.

### Study Design, Research Evidence, and Aim of the Research

Table 2 depicts the assignment of the publications to the levels of research evidence established by Grove [31]. The studies included in our review were mainly allocated to the level VI evidence (10/12, 83%) because most of the studies were descriptive in nature. Moreover, Table 2 shows the aim of the research with respect to the targeted AI- and ML-enabled applications, along with the categories framed by von Gerich et al [45]. As for the aim of the research, most of the included publications dealt with the development of AI- and ML-driven applications (10/12, 83%).

**Table 2:** Study design, level of research evidence, and aim of the research.

Characteristic		Author, year of publication	Publications (n=12), n (%)
**Study design by the level of research evidence**			
	Level I (eg, meta-analysis)	N/A^a^	0 (0)
	Level II (eg, randomized controlled trial)	Kobayashi et al [37,38], 2022	1 (8)
	Level III (eg, quasi-experimental study)	Shorey et al [43], 2023	1 (8)
	Level IV (eg, descriptive correlational study)	N/A	0 (0)
	Level V (eg, qualitative meta-synthesis)	N/A	0 (0)
	Level VI (eg, descriptive study)	Carnell et al [34], 2019; Furlan et al [35], 2021; Hamdy et al [36], 2017; Jani et al [32], 2020; Liaw et al [33], 2023; Maicher et al [39], 2019; Maicher et al [40], 2023; Shorey et al [41], 2019; Shorey et al [42], 2020; Tavarnesi et al [44], 2018	10 (83)
	Level VII (eg, opinions of expert committees and authorities)	N/A	0 (0)
**Aim of the research**			
	To develop AI^b^- or ML^c^-enabled technologies	Furlan et al [35], 2021; Hamdy et al [36], 2017; Kobayashi et al [37,38], 2022; Liaw et al [33], 2023; Maicher et al [39], 2019; Maicher et al [40], 2023; Shorey et al [41], 2019; Shorey et al [42], 2020; Shorey et al [43], 2023; Tavarnesi et al [44], 2018	10 (83)
	To improve the accuracy and efficiency of AI- or ML-enabled technologies	Carnell et al [34], 2019	1 (8)
	To test different algorithms or AI- or ML-enabled technologies	Jani et al [32], 2020	1 (8)
	To assess and evaluate or validate the existing AI- or ML-enabled technologies	N/A	0 (0)

^a^N/A: not applicable.

^b^AI: artificial intelligence.

^c^ML: machine learning.

### Use of AI and ML

In the context of using virtual patients, AI and ML are used to simulate virtual patients who interact with learners. In such cases, AI and ML are used to either predict students’ success in domain-based skills while interacting with virtual patients [34] or generate natural language and natural conversation flow [36,39,41-43]. The second purpose of using AI and ML in the context of communication skills training is to analyze communication data in textual or audio format and provide feedback on them [33,36-39,41-43]. Here, AI and ML automatically scan a text or an audio source for predetermined chunks of information, factors, and aspects. More detailed information on the characteristics of the included studies and their corresponding technological applications is presented in Table 3.

**Table 3:** Characteristics of the included publications.

Category and author, year of publication			Intervention or technological application		Participants and target group		Main outcomes		Key conclusions
**AI^a^ and ML^b^ for text analysis and information extraction**									
	Jani et al [32], 2020	ML as a tool for extracting information and content with respect to communication and history-taking skills in OSCE^c^ transcripts		121 transcripts of 2 OSCE scenarios were analyzed for communication skills and specific communication and history-taking content domains.		The used ML models were able to successfully label the OSCE transcripts for predetermined communication and history-taking factors.		The presented ML-based model represents one of the first applications of ML in the framework of an automatized analysis of medical OSCE conversations. In addition, the ML-based model showed a great degree of transferability across distinct scenarios.	
**AI and ML and virtual reality in communication skills training for students pursuing health care profession education**									
	Liaw et al [33], 2023	An AI-driven virtual reality simulation system, by means of which nursing students can practice interprofessional communication skills with an AI physician		32 nursing students in their final year of nursing courses took part in the study.		The AI-enabled virtual reality simulation system proved effective. Participants showed substantial improvements in communication skills upon practicing with the system.		The AI-driven virtual reality simulation system possesses great potential to advance interprofessional communication skills training in nursing education.	
**AI and ML and virtual patients in communication skills training for students pursuing health care profession education**									
	Carnell et al [34], 2019	Automated communication skills assessment by means of AI and ML and virtual patients. Of note, an interpretable ML model, Bayesian rule lists, was used.		464 transcripts of speech language pathology students’ interactions with virtual patients showing symptoms of dysphagia were analyzed.		The results supported the hypothesis that communication skills and success of an interviewer in a given skill domain (eg, transfer of medical knowledge) are closely linked. The Bayesian rule lists proved competitive in comparison with common ML algorithms.		The used interpretable The ML model, Bayesian rule lists, was shown to be viable in term of the prediction of students’ success in communication skills learning scenarios with virtual patients.	
	Furlan et al [35], 2021	An AI-driven virtual patient simulator for diagnostic reasoning that incorporates natural language processing and an intelligent tutoring system		15 undergraduate medical students in their fifth year at Humanitas University Medical School in Italy who received introductory lectures on the simulator and were able to use it beforehand.		“Hepius,” an AI-enabled virtual patient simulator system, was developed; it provides students with a tool to practice the collection of clinical information from the patient’s medical history and physical examination. By means of the simulator, a differential diagnosis can be reached. In addition, “Hepius” functions as an intelligent tutoring system that is able to provide step-by-step feedback to the student.		The developed virtual patient simulator “Hepius” may provide medical students with a learning instrument for the training of diagnostic reasoning. Most notable is the combination of natural language processing and intelligent tutoring system aspects to provide learners with a comprehensive practice tool.	
	Hamdy et al [36], 2017	AI- and ML-based web-based communication simulation system with virtual patients using prerecorded movies		Students and faculty staff using virtual patient learning for the enhancement of problem-based learning		It is reported from first-hand experience that most students and faculty staff would enjoy using the tool, which would encourage them to be more focused and systematic throughout the medical interview. Students would feel more accountable for their decisions and more involved in deciding how to manage the patient’s problem.		Virtual patient learning is believed to gradually replace problem-based learning in medical communication training.	
	Kobayashi et al [37,38], 2022	A simulated communication skills training for nursing students by means of AR^d^ with real-time AI-driven feedback		25 nursing students participated in the study and were randomly allocated to 1 of 2 conditions: AR-training or conventional nursing mannequin training.		The students who received an intervention with the simulated communication skills training showed an improvement in terms of empathy toward patients, whereas the students in the control condition showed a deterioration in this regard. In addition, the proportion of time spent in eye contact with the patient was shown to be higher for the AR group than for the control group.		The AI-driven communication skills training simulation was associated with increased interactive communication skills and higher empathy toward patience.	
	Maicher et al [39], 2019	An AI-driven virtual standardized patient system that uses emotionally responsive 3D characters that interact with students on the basis of natural language processing		102 first- and second-year medical students volunteered to interview the virtual standardized patient with a complaint of back pain.		Scores calculated by the computer system did not differ from the scores given by the human raters. The overall accuracy of the computer system was 87%, not differing much from the 90% accuracy of the human raters.		The system proved viable and enabled students to practice and receive feedback in an always-accessible risk-free environment.	
	Maicher et al [40], 2023	An AI-enabled virtual standardized patient system consisting of automated speech recognition, 2 distinct AI systems, a classifier to choose between the 2 systems, and automated speech generation		620 first-year medical students of the years 2018, 2019, and 2021, whose data were analyzed.		Conversational accuracy was improved by the hybrid nature of the new AI-driven system.		A novel hybrid AI-driven dialogue system incorporating virtual standardized patients was developed. Learners may use this system to practice and refine their history-taking and communication skills to prepare for real-world scenarios.	
	Shorey et al [41], 2019	A virtual counseling application using AI and a 3D avatar for the training of nursing communication skills		N/A^e^		A virtual counseling application using AI for communication skills training in nursing education was created.		A corresponding application will provide huge, cost-efficient, always-accessible support for communication skills training in health care education. A longitudinal quasi-experimental follow-up study over the course of 2 years was planned.	
	Shorey et al [42], 2020	A virtual counseling application using AI and a 3D avatar for the training of nursing communication skills		24 nursing students (n=6, 25% men and n=18, 75% women) aged 20 to 33 years as well as 6 clinical facilitators (all women) aged 32 to 58 years participated in the study.		Results revealed several benefits and shortcomings of the use of virtual patients in nursing students’ learning.		Student users and clinical facilitators showed positive attitudes toward the AI-based virtual counseling application. Factors that still have to undergo improvements have been identified.	
	Shorey et al [43], 2023	A virtual counseling application using AI and a 3D avatar for the training of nursing communication skills		93 undergraduate nursing students		Practicing with the virtual counseling application improved students’ learning attitudes toward communication skills and increased perceived self-efficacy.		Generally speaking, the developed virtual counseling application may provide learners with a worthwhile and cost-effective way of practicing nursing communication skills.	
	Tavarnesi et al [44], 2018	Introduction to a virtual patient learning system, a web-based simulation system designed to train and assess learning scenarios in medical communication skills education		118 medical students in their second year of the study program and 59 medical tutors completed a survey.		A virtual patient simulator system was developed and established based on prerecorded clips with real actors. The system was specifically designed to improve users’ effectiveness in the areas of anamnesis, diagnosis, treatment, and follow-up.		The virtual patient simulator system showed great potential for enhancing the learning experience and promoting discussion among students and potential for helping with information and content retention.	

^a^AI: artificial intelligence.

^b^ML: machine learning.

^c^OSCE: objective structured clinical examination.

^d^AR: augmented reality.

^e^N/A: not applicable.

### Determinants for the Use of AI and ML in Communication Skills Training for Students Pursuing Health Care Profession Education

Factors that were described as supporting the use of AI and ML in communication skills training were motivated educators, instructional designers, and health care students [35,36,41-44]. The motivation of these agents was especially enhanced through user-friendly interfaces [40,44] or a positive evaluation of the virtual learning environment [33]. Factors that were described as hindering the use of AI and ML in communication skills training were the facts that technological performance lags behind human performance and that the possible uses were restricted because of the complex nature of human communication in health care [32,34,35,39,41-43]. Correspondingly, the experience with virtual patients was described as “not meaningful,” among others, owing to the limitation and predictability of the virtual patient’s responses [43]. It was also reported that practicing with the virtual patient system required a higher degree of self-discipline and enthusiasm compared with conventional communication skills training [32,40,43]. In addition, the implementation of AI- and ML-driven systems is accompanied by a requirement for a certain investment in terms of time and cost, which may pose a barrier [32,35,40]. Table 4 shows the enablers of and barriers to the use of AI and ML in communication skills training for health care professionals in more detail.

**Table 4:** Supporting and hindering factors for the use of artificial intelligence (AI) and machine learning (ML) in communication skills training for students pursuing health care profession education.

Author, year of publication	Supporting factors	Hindering factors
Carnell et al [34], 2019	No information	The use of AI and ML to predict medical interviewing competencies is currently limited to a small array of conversational features.
Furlan et al [35], 2021	Especially in times such as the current pandemic, the need for automated clinical training methods designed for distant learning functions as a fostering factor for the implementation of ML-based simulator systems such as “Hepius” and increases the motivation of the involved staff.	A potential barrier may be imposed by the learning curve typical to “Hepius.” Users first have to get accustomed to the system before its full potential can be developed.
Hamdy et al [36], 2017	The motivation of the students and faculty who used the tool was a facilitating factor for the widespread implementation of the VPL^a^ system.	No information
Jani et al [32], 2020	No information	Despite the circumstance that ML could potentially reduce training costs and provide a time-efficient method of practice, at first, it comes with its own costs in terms of development, deployment, and implementation.
Kobayashi et al [37,38], 2022	No information	No information
Liaw et al [33], 2023	The acceptability, feasibility, and usability of the AI-driven virtual reality simulation were favorable based on students’ positive evaluations of the virtual learning environment. The virtual reality simulation was especially approved by students if it was offered as an alternative to the existing practicing modalities, particularly amid the COVID-19 pandemic. There is a need to build and integrate agents controlled by computer algorithms because of unequal cohort sizes across different health care courses (eg, 300 medical students vs 1500 nursing students), which impede the creation of interprofessional teams to engage in physician-nursing team training.	No information
Maicher et al [39], 2019	No information	The current system is restricted to the evaluation of information within a specific domain. Various factors such as empathy, nonverbal communication, and physical examinations are currently not controlled for. In addition, the system lacks flexibility.
Maicher et al [40], 2023	No information	Usually, for an ML model to perform well, it needs adequate and comprehensive training. This may pose as a barrier in terms of time and cost.
Shorey et al [41], 2019, and Shorey et al [42], 2020	The development and introduction of the virtual patient learning system was boosted by highly motivated staff and stakeholders who found the traditional methods of communication skills training (eg, employing standardized patients) too expensive and resource demanding.	The virtual patient system was incapable of adapting to the context of the conversation; acting authentically on conversational intentions that went beyond the guidelines, such as indulging in small talk and providing advice; and showcasing a natural flow of language.
Shorey et al [43], 2023	No information	Using the virtual patient simulation system came along with unique challenges due to the limitations and predictability of the virtual patient’s responses. Furthermore, the experiences with the virtual patient were described as “not meaningful.” Finally, it was reported that practicing with the virtual patient system required a higher degree of self-discipline and enthusiasm compared with conventional communication skills training.
Tavarnesi et al [44], 2018	The successful implementation of the AI- and ML-based learning system depended on whether the interface was perceived as user-friendly (eg, “clear and clean” and “no doubts about how to start the simulator...”), which, in turn, led the learners to rate the system as enjoyable and increased their motivation for use it.	Current barriers revolve around the fact that the AI- and ML-based virtual patient system is limited to specific cases, topics, guidelines, patient personalities, relational styles, outcomes, and clinicians (eg, nurses, pharmacists, and caregivers).

^a^VPL: virtual patients learning.

## Discussion

### Principal Findings

This scoping review provides a comprehensive overview of the status quo regarding the application of AI- and ML-enabled technologies in the communication skills training in health care profession education. Most studies at this point in time deal with the development of AI- and ML-driven systems, showing once again that these technologies are still in their infancy. The most frequently drawn conclusions in the examined publications were that AI and ML possesses great potential in the field under consideration. The interventions were predominantly evaluated as working as intended. Enabling factors such as motivation and barriers such as technological limitations are to be considered on the path from the blueprint to the practice.

### How Can AI Support the Training of Communication Skills?

Many of the included publications addressed the use of AI and ML to generate natural language within the context of virtual patient conversations [36,39,41-44]. The evidence collected rated this use of AI and ML in communication skills training as viable and highly promising because it has the potential to create various training scenarios that can be customized to specific real-world situations [39-43]. Learners may greatly benefit from practicing simulated patient encounters that are tailored to the challenges they face in their everyday clinical practice. This approach emphasizes the role of linguistic authenticity in the context of communication skills training for medical professionals using virtual patient systems, as the authenticity of speech and language was identified as a core component of a successful patient encounter with AI- and ML-simulated virtual patients [36,39-44]. However, according to the current state of research that we investigated, the generation of natural and authentic language still poses a huge challenge in the introduction of virtual patients to communication skills training [36,39-44,46,47]. This may be a starting point for further research.

The second main finding was that AI and ML may be efficiently used for text analysis, processing spoken language, and identifying prespecified factors in recorded patient-health care provider conversations [36,39,41-43]. At this point in time, it is already possible for an AI or ML system to scan spoken language or written text (eg, transcripts) for predetermined aspects and pieces of information [32,37,38]. Thereby, the examined communication can be evaluated, and feedback can be provided to the learner [36-39,41-43]. In addition to this practical use, the AI or ML system itself may be trained to give the learner feedback instead of having a third party be involved in the process, thus saving time and resources. This is especially helpful for repeated run-throughs of cases that are associated with a higher risk of potential malpractice claims [39,48]. In addition, a learner may hesitate to reach out to a human trainer or supervisor owing to limited time or possible stigma involved in seeking help [49], both of which can be addressed by using an AI and ML system. Although AI- and ML-based text and speech analysis systems are still in the early stages of their implementation, they are already considered beneficial in everyday practice by many [48,50]. However, although several natural language processing systems specifically developed for the health care domain are available [51], they are predominantly used in areas such as pharmacogenomics, diagnosis categorization, and novel phenotype discovery [52]. Therefore, a prospective next step in the field of communication skills training for health care professions would be to further apply these systems to the realm of communication skills education. One of the possible and already practiced ways of introducing natural language processing systems to communication skills training for health care professions is the aforementioned generation of natural language in the context of virtual patient simulations.

In conclusion, both the generation of natural language in the context of simulated virtual patient-health care provider encounters and the AI- and ML-based analyses of language and speech to provide learners with feedback were evaluated as viable and promising for practical use. On the basis of this assessment, both approaches to using AI and ML in communication skills training should be upheld and are recommended to be further integrated and disseminated in communication skills training for students pursuing health care profession education. The combination of both methods can already be found in practice in the form of virtual patients whose communication with students is mainly based on natural language processing systems [41-43,49]. Further steps toward a broad-scale implementation would include corresponding analyses of the demand for these systems, the use of specific interventions when introducing these systems, and an ongoing summative evaluation of their use [53,54].

### Determinants for the Use of AI and ML in Communication Skills Training

One aspect worth mentioning in relation to the enablers is the factor of motivation. It can be derived from the literature included in this review that motivation can be considered the most important factor in the implementation of AI- and ML-based systems in communication skills training for health care professions [35,36,41-44]. This is particularly interesting, as the ongoing COVID-19 pandemic as well as the general digitalization in health care provides incentives to further implement new technologies such as AI [33,35]. In line with this reasoning, the use of AI and ML is politically broadly endorsed [55]. Furthermore, learners’ motivation to use AI- and ML-enabled systems seems to be influenced by whether they evaluate the virtual simulation environment as positive [33] or the system as user-friendly [44].

With regard to the hindering factors and especially in light of the current developments and breakthroughs in the domain of AI and ML such as ChatGPT [56], it should be noted that the technological performance of AI- and ML-based communication tools and conversation simulation systems still seems to lag behind human performance [32,34,35,39,41-44]. Of course, AI- and ML-based technologies vary in their functions as communicative unit [46], thus rendering general statements about AI- and ML-based technological performance rather vague. After all, the nature of human expression within a conversation with its functional as well as relational dimensions and dynamics still constitutes an obstacle for AI- and ML-based communication systems [43,46,47]. This is in line with users of virtual simulation systems describing their encounters with AI-driven patients as “not meaningful,” as the responses of these patients are as limited as predictable [43]. Finally, the implementation of AI- and ML-enabled systems requires an investment of time and cost, which is not to be underestimated [32,35,40].

### General Findings

Our scoping review discovered that the current focus in terms of the use of AI and ML in the area of undergraduate communication skills training in health care profession education is placed on medical [32,35,36,39,40,44] and nursing students [33,37,38,41-43]. Students pursuing education in other health care professions were targeted by only 1 (8%) of 12 included studies, namely speech language pathology students [34]. This may be associated with the absolute number of health care workers who are foremost physicians and nurses [57] and the accompanying long tradition in education and research in these 2 professions in general and in regard to patient-health care provider communication [58].

Of the 12 included studies, 1 (8%) did not focus on patient-health care provider communication but on interprofessional nurse-physician communication [33]. This approach seems especially promising in terms of the reported barriers to interprofessional education [59]. AI agents in virtual reality that substitute other professions in the training scenario respond to the institutional barriers of interprofessional education, for example, the scheduling of interprofessional education initiatives with different course timetables, condensed curricula, and different cohort sizes, which was mentioned by Liaw et al [33] as a motivation for their innovative approach in virtual reality. Such training programs might also reduce barriers on a personal level because biases or a lack of respect toward one’s own profession or a higher workload can be considered in the development phase.

A potential transfer of the AI- and ML-driven applications to be used in other health professions would have to be mainly based on the analyses of the demand for these systems within the specific health care domain as the current demand for AI- and ML-based systems in other health care professions is yet to be determined [60,61]. Once a specific need has been identified, a tailored intervention to implement a designed system or application would have to be developed. In conclusion, a continuous summative evaluation during implementation will ensure a successful implementation [54,62]. From a technical point of view and as deduced from the examined literature, a transfer of AI- and ML-based virtual patient simulation systems to other health care areas would be viable because the use of AI- and ML-based virtual patients is heavily based on learning specific vocabulary for a prespecified scenario [32,44]. Accordingly, it is considered possible to teach AI- and ML-based systems different vocabulary, that is, for a different scenario.

### Web-Based Consultations

Current literature suggests an increasing demand for web-based consultations [63-66]. On the basis of this evidence, it is reasonable and efficient to equip undergraduates in communication skills training for health care professionals with the possibility to practice with virtual patient-health care provider encounters. Accordingly, with the rise in video consultations, the use of simulated virtual patients may gain additional legitimacy, as it provides learners with a cost-efficient and readily available means to practice video consultations, in accordance with the best practices for the provision of virtual care [67].

### Comparison With Prior Work

As the last reviews broaching the area of AI-supported communication skills training [48,49], the use of AI and ML in the communication skills training of students pursuing health care profession education has proven to possess great potential in several aspects. As proposed by Ryan et al [46], AI and ML could successfully be implemented in the analytics of communication with the possibility of providing learners with routine feedback on their skills. Specifically, methods for analyzing factors such as turn-taking, pronunciation, and the identification of prespecified keywords have been developed and brought to use. In addition, caretakers as well as trainers and communication professionals have been found to accept the implementation of AI and ML in the feedback processes of communication skills training in health care profession education. In comparison with Butow and Hoque [49], it is especially interesting that most uses of AI and ML in the domain of communication skills training are still limited to a few specific concepts and training cases. Expanding the use of AI and ML to other domains is yet to be done. Furthermore, it remains important to generate evidence for the feasibility, reliability, acceptability, and effectiveness of the implementation of AI and ML in the area of communication skills training. However, some studies on this matter have already been conducted.

### Levels of Research Evidence

Within the framework of this scoping review, we could only identify 1 RCT in the current literature that addressed the use of AI and ML in the communication skills training for patient-health care provider encounters [37,38]. Most of the included studies were of descriptive nature [32-36,39-44]. This may be because AI- and ML-based technologies are still in their infant stage when it comes to their transfer into common practice in health care professions, specifically in medical education [49,62,68]. As these modern technologies and novel systems are advancing and progressively finding their way into direct use and practice, the number of RCTs is estimated to increase [32]. The evaluation of particular uses upon broad-scale implementation may provide fruitful soil for RCT-focused research within this realm. In conclusion, the quality of evidence regarding the effects of AI- and ML-supported communication skills training on the communication skills of undergraduate health care students is relatively low and needs improvement to make recommendations about the use of AI-supported communication skills training.

### Ethical and Epistemological Considerations in the Application of AI-Based Systems

As derived from the latest research, the use of AI-based systems in medicine may come with a certain risk of bias with regard to ethical and epistemological factors [68,69]. Such factors as well as the consideration of the intelligibility and accountability of AI-based medical technology are not covered sufficiently in the current literature, that is, the publications included in this review. This may also entail a barrier to the implementation of AI-based systems, as opaque algorithms, often referred to as black box, are widely considered unacceptable [70]. An exception to this is constituted by the study by Jani et al [32], which discusses these aspects and implications for humanity in medicine in the discussion section. Carnell et al [34] acted on these considerations by explicitly using interpretable AI models.

### Limitations

With regard to the limitations of this scoping review, different terms or synonyms may have been used in the existing literature to describe AI and ML systems [49,68]. Therefore, there may be relevant studies that our search strategy could not identify and, therefore, were not included in this review.

Another limitation may be constituted by the circumstance that this review deals with undergraduate health care education. Accordingly, any research that was conducted on postgraduate level of health care education was excluded from this work. However, the AI- and ML-driven interventions and applications used in these studies may potentially be adapted to undergraduate education to enhance the richness of feedback on communication skills and on the performance in workplace-based assessments [71,72]. The same applies to AI- and ML-enabled text analysis systems that are currently being researched but have not been applied to the context of communication skills training yet [73-75].

Finally, the term *artificial intelligence* has recently become more popular, evolving around an abundance of varying definitions and wordings [45,68]. Older publications using outdated terminologies or technologies that we consider as AI without naming it *AI* may, therefore, not have been identified by our search.

### Conclusions

In conclusion, our scoping review identified 2 main forms of AI- and ML-driven applications that are currently used in the field of communication skills training in the health care profession education: the AI- and ML-driven simulation of virtual patients to provide learners with a tool to practice communication and the AI- and ML-driven analysis of communication by means of text or audio to provide learners with feedback on their communication performance. In the process of implementing these technologies, motivation plays a major role as an enabler, whereas the fact that technological performance still lags behind human performance in most cases, especially when it comes to the authenticity of patient simulations, is considered a hinderance.

### Future Works

The possibility to provide learners with a cost-efficient and personalized option for practicing communication skills that is readily available, gives feedback immediately after the conversation with a simulated realistic virtual patient, and is based on natural language processing seems highly promising. As the quality of such simulated encounters highly depends on whether they are perceived as authentic, future research may want to focus more specifically on the generation of natural and authentic language in the context of the aforementioned way of using AI and ML. Furthermore, the current implementation science may be used to appropriately and efficiently implement the existing and upcoming AI- and ML-driven systems in practice. More RCTs are needed to make assumptions about the effectiveness of AI-supported communication training.

## Appendix

Multimedia Appendix 1 PRISMA-ScR (Preferred Reporting Items for Systematic Reviews and Meta-Analyses extension for Scoping Reviews) checklist.

Multimedia Appendix 2 Complete search strategy.

## Abbreviations

AI: artificial intelligence
ML: machine learning
PRISMA-ScR: Preferred Reporting Items for Systematic Reviews and Meta-Analysis extension for Scoping Reviews
RCT: randomized controlled trial
